# Age-related STING suppression in macrophages contributes to increased viral load during influenza a virus infection

**DOI:** 10.1186/s12979-024-00482-9

**Published:** 2024-11-14

**Authors:** Thurid Lauf, Antje Häder, Franziska Hornung, Yasmina Reisser, Sandor Nietzsche, Fabian Schanz, Verena Trümper, Aldona Jeznach, Sascha Brunke, Torsten Doenst, Tomasz Skirecki, Bettina Löffler, Stefanie Deinhardt-Emmer

**Affiliations:** 1 Institute of Medical Microbiology, Jena University Hospital, Jena, Germany; 2 Else Kröner Graduate School for Medical Students “JSAM”, Jena University Hospital, Jena, Germany; 3 Center for Electron Microscopy, Jena University Hospital, Jena, Germany; 4 Department of Microbial Pathogenicity Mechanisms, Hans-Knöll-Institute, Jena, Germany; 5 Department of Translational Immunology and Experimental Intensive Care, Centre of Postgraduate Medical Education, Warsaw, Poland; 6 Klinik für Herz- und Thoraxchirurgie, Jena University Hospital, Jena, Germany

**Keywords:** Influenza A virus, Mitochondria, cGAS-STING pathway, Ageing, Macrophages

## Abstract

Ageing is a major risk factor that contributes to increased mortality and morbidity rates during influenza A virus (IAV) infections. Macrophages are crucial players in the defense against viral infections and display impaired function during ageing. However, the impact of ageing on macrophage function in response to an IAV infection remains unclear and offers potential insight for underlying mechanisms. In this study, we investigated the immune response of young and aged human monocyte-derived macrophages to two different H1N1 IAV strains. Interestingly, macrophages of aged individuals showed a lower interferon response to IAV infection, resulting in increased viral load. Transcriptomic data revealed a reduced expression of stimulator of interferon genes (STING) in aged macrophages albeit the cGAS-STING pathway was upregulated. Our data clearly indicate the importance of STING signaling for interferon production by applying a THP-1 STING knockout model. Evaluation of mitochondrial function during IAV infection revealed the release of mitochondrial DNA to be the activator of cGAS-STING pathway. The subsequent induction of apoptosis was attenuated in aged macrophages due to decreased STING signaling. Our study provides new insights into molecular mechanisms underlying age-related immune impairment. To our best knowledge, we are the first to discover an age-dependent difference in gene expression of STING on a transcriptional level in human monocyte-derived macrophages possibly leading to a diminished interferon production.

**Supplementary Information:**

The online version contains supplementary material available at 10.1186/s12979-024-00482-9.

## Introduction

Influenza A virus (IAV)-induced pneumonia is an infection of the lower respiratory tract associated with high mortality and morbidity rates worldwide [1]. A main risk factor for developing a severe course of disease is age [2]. Of all deaths associated with IAV infections, more than two-third happen in patients aged ≥ 65 years [3]. In severe cases, the disruption of the alveolar-capillary barrier can results in a massive infiltration of immune cells ultimately leading to an acute respiratory distress syndrome (ARDS) [4].

Here, the innate immune system serves as the first line of defense against invading pathogens, providing a rapid and non-specific response to infections. In particular alveolar macrophages (AM) reside directly at the air-tissue interface and recognize pathogen-associated molecular patterns (PAMPs) with pattern recognition receptors (PRRs). Upon virus infection, AM produce interferons (IFN) to restrict viral replication especially in the early stages of infection as well as other pro-inflammatory cytokines and chemokines such as tumor-necrosis factor α (TNFα), interleukin 6 (IL-6) and interleukin 8 (IL-8) [5]. The inflammation attracts circulating monocytes to the lung where they differentiate to macrophages and contribute to the antiviral immune response [6, 7]. Interestingly, besides their role in protection against IAV infection, macrophages may also become infected and support virus replication. The ability of IAV to successfully replicate in lung macrophages is discussed controversially in literature and is dependent both on the IAV strain and macrophage subset [8, 9].

A crucial component for a sufficient immune response in AM are mitochondria. Upon activation, immune cells undergo metabolic shifts to increase energy demands associated with effector functions [10]. Here, mitochondria play a central role in regulating these metabolic changes, influencing cellular processes such as glycolysis, oxidative phosphorylation, and fatty acid oxidation [11].

Furthermore, mitochondria are important in modulating antiviral signaling pathways. The mitochondrial antiviral signaling protein (MAVS) is located downstream of retinoic acid-inducible gene-I (RIG-I)-like receptors (RLRs) which are specialized receptors for the recognition of viral RNA [12]. MAVS activates the transcription factor interferon regulatory factor 3 (IRF3) and induces production of type-I interferons [13]. Additionally, MAVS initiates localization of NLRP3 to mitochondria and activation of inflammasome response [14]. During ageing, mitochondrial dysfunction is reported in a variety of cells and contributes to age-related changes of metabolism and inflammation [15].

It is known that the dysfunction of mitochondria leads to the release of mitochondrial DNA (mtDNA) into the cytoplasm, leading to the activation of cyclic GMP-AMP synthase (cGAS)-stimulator of IFN gene (STING) pathway [16, 17]. Also the infection with IAV stimulates the release of mtDNA and subsequently the activation of the cGAS-STING axis [18].

In our study, we aimed to investigate the effect of ageing in macrophages with particular focus on mitochondria dysfunction. By infection with different IAV strains in vitro and by employing of a human ex vivo lung model, new insights into age-related changes of macrophage function were obtained. Our data provides new insights into age-related changes of macrophage function caused by differences in cGAS-STING signaling and mitochondrial dysfunction during IAV infection.

## Results

### Reduced interferon response and elevated virus titers in macrophages of elderly individuals after infection with a IAV/PR8 strain

**Fig. 1:**
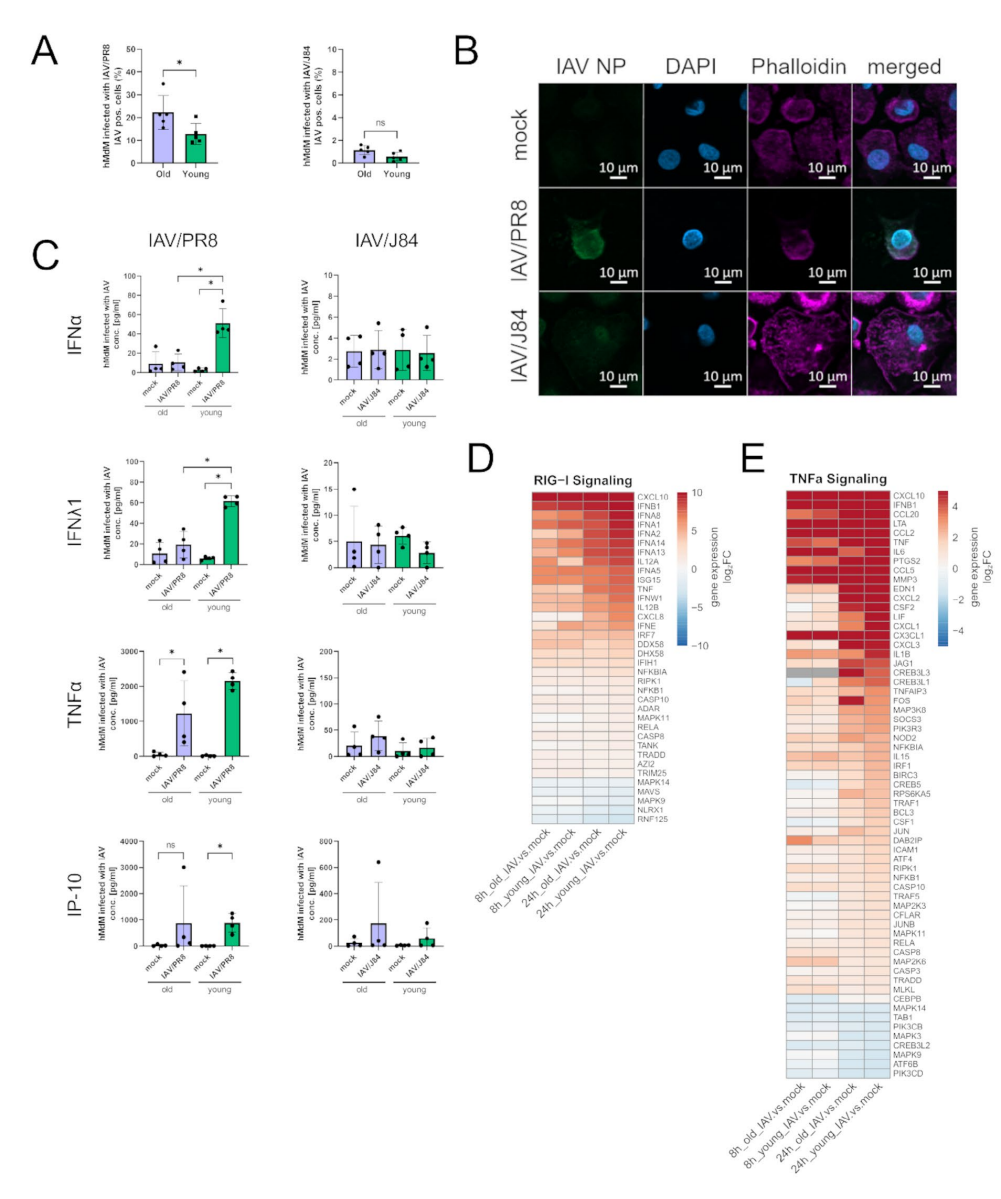
Suppression of interferon response and increased susceptibility to infection in macrophages of elderly individuals. **(A)** Measurement of virus-positive hMdM 24 h p.i. after infection with IAV (MOI 1). The experiment was conducted with five young and old donors each. Each data point represents a biological replicate in this and all following figures. Significance was calculated with the Mann-Whitney U test (* *p* ≤ 0.05). **(B)** Immunofluorescence staining of infected hMdM (24 h p.i.) with antibody against IAV NP, phalloidin and DAPI. **(C)** Cytokine concentrations of IFNα, IFNλ1, TNFα and IP-10 were measured in the supernatant of infected hMdM (24 h p.i.). The results of four young and old donors each are shown. Significance was calculated with the Mann-Whitney U test (* *p* ≤ 0.05). **(D)** DEGs are shown in heatmaps based on KEGG pathways regarding RIG-I signaling, and **(E)** TNFα signaling at timepoint 8 h and 24 h p.i. Gene expression in heatmaps over log2 fold change. Red signals upregulation, blue downregulation. The DEG of at least one condition per row had a log2 fold change > 1 with a significance of *p* ≤ 0.05

To investigate susceptibility of macrophages to IAV infection we infected hMdM from young (≤ 25 years, refers as young macrophages) and aged (≥ 65 years, refers as aged macrophages) donors with a laboratory IAV/PR8 H1N1 (IAV/PR8) and contemporary H1N1 (IAV/J84) IAV strain isolated in 2016 and measured virus-positive cells via flow-cytometry after 24 h (Fig. 1A, Supplemental Fig. 1A, B). Interestingly, only the IAV/PR8 strain was able to infect and successfully replicate in hMdM. By using immunofluorescence staining with antibodies against IAV Nucleoprotein (NP), we confirmed intracellular localization of viral proteins solely after infection with IAV/PR8 (Fig. 1B).

To further investigate the effect of age on the replication, we performed IAV infection in old and young macrophages. Interestingly, the aged hMdM displayed a significantly higher susceptibility to infection compared to young hMdM (Fig. 1A). To analyse whether this effect is accompanied with an impaired inflammatory response, we measured cytokines in the supernatants of infected hMdM. Aged hMdM produced significantly less IFNα and IFNλ1 after infection with IAV/PR8 (Fig. 1C). Other antiviral and inflammation markers such as IP-10 and TNFα were also elevated after infection with IAV/PR8, however with no difference between aged and young hMdM (Fig. 1C). All hMdM displayed no significant cytokine response to infection with the contemporary H1N1 strain (Fig. 1C).

To explore underlying mechanisms of the age-dependent impairment in interferon production, we performed mRNA sequencing of young and aged hMdM infected with IAV/PR8 at timepoints 8 h and 24 h post infection (p.i.) (Supplemental Fig. 1B-E). KEGG Pathway enrichment analysis showed that genes related to the RIG-I and TNFα signaling pathways were significantly enriched (Supplemental Fig. 1E). Differentially expressed genes (DEGs) belonging to those pathways are shown in the heatmaps (Fig. 1D and E). Type-I IFN genes are upregulated in young and aged hMdM infected with IAV/PR8 as early as 8 h p.i.; however, 24 h p.i. they are more strongly upregulated in young hMdM (Fig. 1D). Other inflammatory marker genes such as *IL6* and *IL1B* are also more highly expressed in young hMdM compared to aged hMdM (Fig. 1E).

Our findings indicate an impairment in the inflammatory response of aged hMdM after infection with IAV/PR8 contributing to an increased susceptibility to infection. Additionally, gene expression of interferons and inflammatory markers were reduced in aged hMdM after virus infection.

### STING is downregulated in aged macrophages while the cGAS-STING pathway was upregulated during infection

**Fig. 2:**
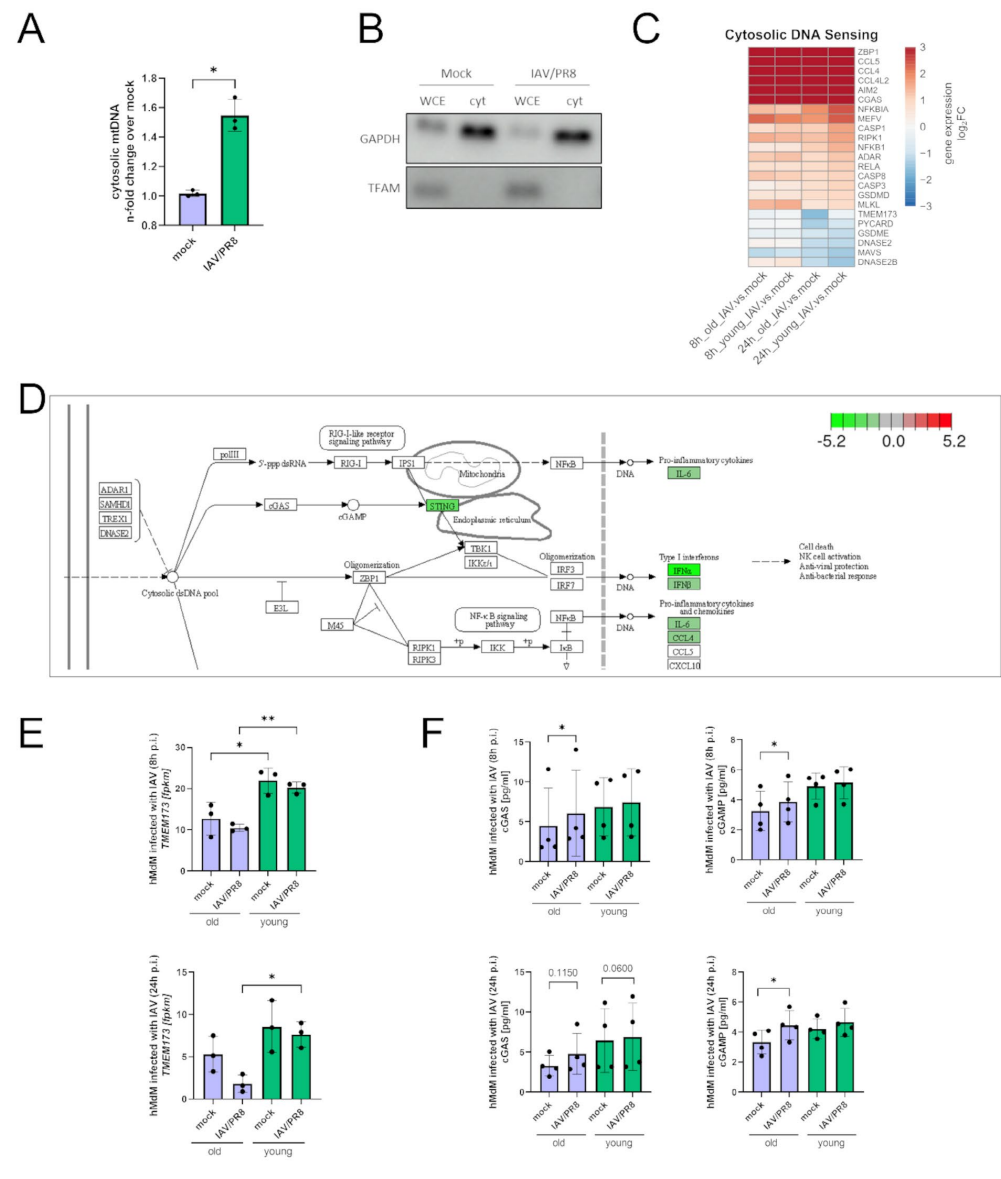
Reduced expression of STING in aged macrophages albeit the cGAS-STING pathway was upregulated during infection. **(A)** Abundance of cytosolic mtDNA 8 h p.i. was measured in three biological replicates of hMdM with quantitative RT-PCR. After infection with IAV/PR8 hMdM show elevated levels of cytosolic mtDNA. Significance was evaluated with paired t-test (* *p* ≤ 0.05). **(B)** Whole cell extracts (WCE) and cytosolic fractions were analyzed by western blot using the indicated antibodies. **(C)** Heatmap with DEGs related to cytosolic DNA sensing pathways. Gene expression is presented over log2 fold change. The DEG of at least one condition per row had a log2 fold change > 1 with a significance of *p* ≤ 0.05. **(D)** KEGG-Enrichment pathway of cytosolic DNA sensing pathway comparing aged hMdM to young hMdM infected with IAV/PR8 24 h p.i. showing reduced expression of STING in aged hMdM. Red signals upregulation, green signal downregulation. **(E)** Gene expression of *TMEM173* was shown as a bar plot. Significance was calculated with Kruskal-Wallis test (* *p* ≤ 0.05). **(F)** Concentration of cGAS and cGAMP were measured with ELISA in four biological replicates of hMdM 8 h and 24 h p.i. Significance was evaluated with paired t-test (* *p* ≤ 0.05)

Our data revealed that there was a significant increase in the abundance of mtDNA in the cytosol of hMdM 8 h after infection with IAV/PR8 (Fig. 2A). We have confirmed the absence of mitochondrial DNA in the cytosolic fraction by western blotting (Fig. 2B). Since it has been shown that during infection with IAV the cGAS-STING pathway is being activated due to release of mitochondrial DNA (mtDNA) [17], we analyzed the genes of the KEGG cytosolic DNA sensing pathway. Here, an enrichment among the differentially regulated genes in infected hMdM was detectable (Supplemental Fig. 1E).

Intriguingly, while the cytosolic DNA sensor cGAS was upregulated in hMdM, STING (*TMEM173*) was downregulated after infection with IAV/PR8 in old macrophages (Fig. 2C). Furthermore, the overall gene expression of STING was reduced in aged hMdM compared to young hMdM infected with IAV/PR8 at both timepoints, 8 h and 24 h p.i. (Figure 2D and E). To further explore the activation of the cGAS-STING pathway, we measured protein levels of cGAS and cGAMP in infected aged and young hMdMs. The concentration of cGAS was significantly increased 24 h p.i., and there was a significant increase of cGAMP in aged hMdM 8 h and 24 h after infection with IAV/PR8 (Fig. 2F).

Combining these findings, our data suggest an activation of the cGAS-STING pathway in hMdM after infection with IAV due to the release of mtDNA. STING expression is decreased after infection; however, aged hMdM display an overall lower expression of STING.

### STING is necessary to induce an interferon response in macrophages

**Fig. 3:**
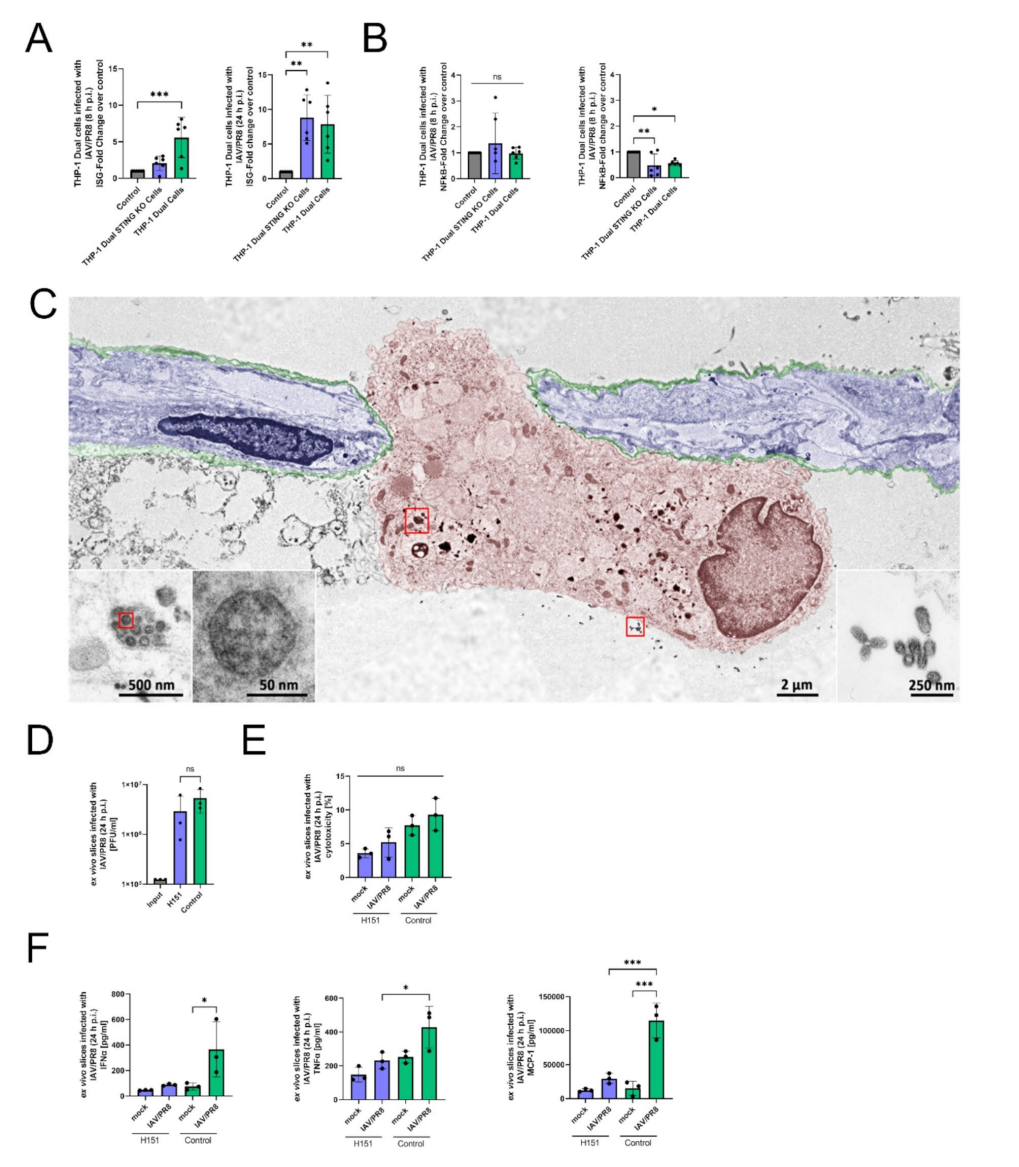
STING is necessary to induce interferon response in macrophages. **(A)** IFN stimulator gene (ISG) reporter fold change of THP-1 Dual and THP-1 Dual STING KO cells infected with IAV/PR8 at timepoints 8 h p.i. and 24 h p.i. The results of six biological replicates of each cell line is shown. Significance was calculated with One-way ANOVA (* *p* ≤ 0.05, ** *p* ≤ 0.01, *** *p* ≤ 0.001). **(B)** Transcriptional activity of NFκB pathway in THP-1 Dual and THP-1 Dual STING KO cells infected with IAV/PR8. Significance was calculated with One-way ANOVA (* *p* ≤ 0.05, ** *p* ≤ 0.01). **(C)** SEMpicture showing IAV on the surface and intracellular of an alveolar macrophage. Colors were added manually. **(D)** Viral titer of ex vivo lung slices infected with IAV/PR8 24 h p.i. was detected by standard plaque assay shown with logarithmic scaling. The results of three biological replicates are shown. Significance was calculated with One-way ANOVA (ns *p* ≥ 0.05). **(E)** LDH was measured in the supernatants of ex vivo slices and presented as percentage of the positive control. Significance was calculated with One-way ANOVA (ns *p* ≥ 0.05). **(F)** Cytokines IFNα, TNFα and MCP-1 were measured in the supernatants. Significance was calculated with One-way ANOVA (* *p* ≤ 0.05, ** *p* ≤ 0.01, *** *p* ≤ 0.001)

To elucidate the importance of STING in the production of interferons, we used a THP-1 Dual STING KO cell model. These cell lines stably express a secreted fetal alkaline phosphatase (SEAP) and a secreted luciferase under the control of IFN and NFκB-responsive promotors (Supplemental Fig. 2A). The measurement of reporter proteins in the supernatants allows observation of transcriptional activity of the IFN and NFκB pathways over time. Our data shows an significant upregulation 8 h p.i. in the control cells infected with IAV/PR8, but not in the STING KO cells, whereas 24 h p.i. the activity reached the same level (Fig. 3A). Transcription activity of NFκB pathway was significantly downregulated 24 h p.i. with IAV/PR8 independent of the STING KO status (Fig. 3B).

To examine the mechanism in a more complex model, we treated human ex vivo lung slices with the specific STING inhibitor H151 and subsequently infected them with IAV/PR8 (Supplemental Fig. 2B). In order to visualize a successful infection of AM in human ex vivo lung slices, scanning electron microscopy (SEM) was performed (Fig. 3C). Here, we could visualize IAV particles inside of an AM indicating a successful infection (Fig. 3C). Additionally, we performed immunofluorescence staining of the ex vivo slices picturing intracellular IAV NP in AM (Supplemental Fig. 2C).

The cytotoxicity measured in the supernatants was slightly decreased in those slices treated with H151 (Fig. 3E). However, concentrations of IFNα, TNFα and monocyte chemoattractant protein-1 (MCP-1) were decreased in the supernatants of slices treated with H151 (Fig. 3F). The ex vivo full lung model indicates that the diversity of lung cells with their differential regulation and the distinct virus replication in the fibroblasts and especially lung epithelial cells can result in a distortion of the results.

Overall, the in vitro findings demonstrate that STING is necessary to induce an interferon and inflammatory response to IAV infection.

### Mitochondria undergo oxidative stress during infection

**Fig. 4:**
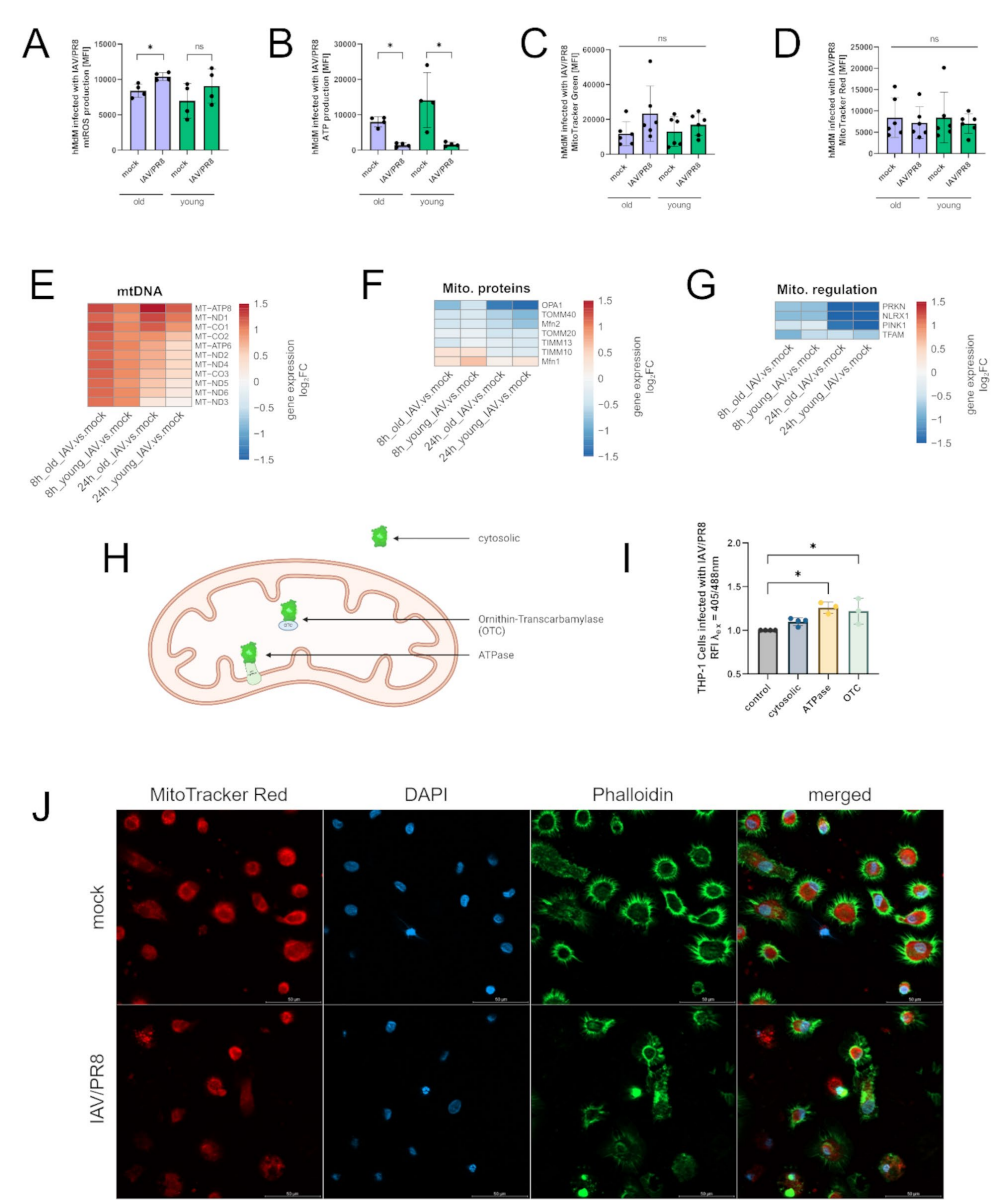
Mitochondria undergo oxidative stress during infection. **(A)** mtROS production in infected hMdM was measured via flow cytometry. The results of four biological replicates are shown. Significance was calculated with the Mann-Whitney U test (* *p* ≤ 0.05). **(B)** Mitochondrial ATP was quantified using flow cytometry. Significance was calculated with the Mann-Whitney U test (* *p* ≤ 0.05). **(C)** Mitochondrial mass in hMdM after infection was measured via staining with MitoTracker Green. The results of six biological replicates are shown. Significance was calculated with the Mann-Whitney U test (ns *p* ≥ 0.05). **(D)** Mitochondrial membrane potential was quantified by staining with MitoTracker Red and measurement via flow cytometry. Significance was calculated with the Mann-Whitney U test (ns *p* ≥ 0.05). **(E)** Heatmaps showing individually differentiated genes related to regulation of mitochondrial metabolism, **(F)** imported mitochondrial proteins and **(G)** regulation of mitochondrial homeostasis. Gene expression is presented over log2 fold change. At least one DEG per gene per condition had a significant log2 fold change. **(H)** Schematic representation of THP-1 redox cell model showing subcellular location of different roGFP2 constructs. **(I)** Determination of redox status of THP-1 cells stably expressing cytosolic roGFP2, roGFP2 coupled to mitochondrial ATPase and OTC. Redox status was analyzed by measuring fluorescence at *λ*ex 405 nm and 488 nm. RFI is normalized to control cells. Significance was calculated with Kruskal-Wallis test (* *p* ≤ 0.05). **(J)** Immunofluorescence staining of infected hMdM stained with MitoTracker Red, phalloidin and DAPI

Since leakage of mtDNA is characteristic for stressed mitochondria, we aimed to characterize mitochondrial functionality during IAV infection. For this, we measured mtROS production in infected hMdM via flow cytometry and in aged hMdM there was a significant increase of mtROS production after infection with IAV/PR8 (Fig. 4A). Additionally, an impaired mitochondrial function results in a decrease in ATP production. To prove this, the production of mitochondrial ATP was measured after infection. We could identify a significant decrease of ATP in young and aged hMdM (Fig. 4B). We proceeded to measure the mitochondrial mass and membrane potential by staining with MitoTracker Green and MitoTracker Red respectively; however, there were no changes during infection (Fig. 4C and D).

By analyzing transcriptomic data, we could observe a general upregulation of genes representative of the mitochondrial metabolism, although the effect was stronger in old hMdM (Fig. 4E). Contrary, genes that encode for mitochondrial proteins that are being imported to the mitochondria and proteins regulating mitochondrial homeostasis were downregulated after infection (Fig. 4F, G).

To further characterize oxidative events during IAV infection, we used THP-1 cell lines stably expressing redox-sensitive green fluorescent protein 2 (roGFP2) in the cytoplasm and coupled to signal sequence of the Subunit 9 of the mitochondrial ATPase and the Ornithin-Transcarbamylase (OTC) (Fig. 4H). The roGFP2 changes its spectral properties dependent on the oxidative milieu, which can be calculated as relative fluorescence intensities (RFI) by using the ratio 405/488 nm. An increase of the ratio compared to control cells was interpreted as a more oxidative milieu. After infection with IAV/PR8 there was a significant increase in the ratio of the mitochondrial markers but not the cytosolic marker (Fig. 4I).

By staining infected hMdM with MitoTracker Red, we could observe a disruption of the mitochondrial network in the infected samples (Fig. 4J). Taken together, our data suggest a disturbance in the mitochondria function caused by the infection with a pandemic IAV strain.

### Apoptosis and inflammasome signaling pathways are altered in old macrophages

**Fig. 5:**
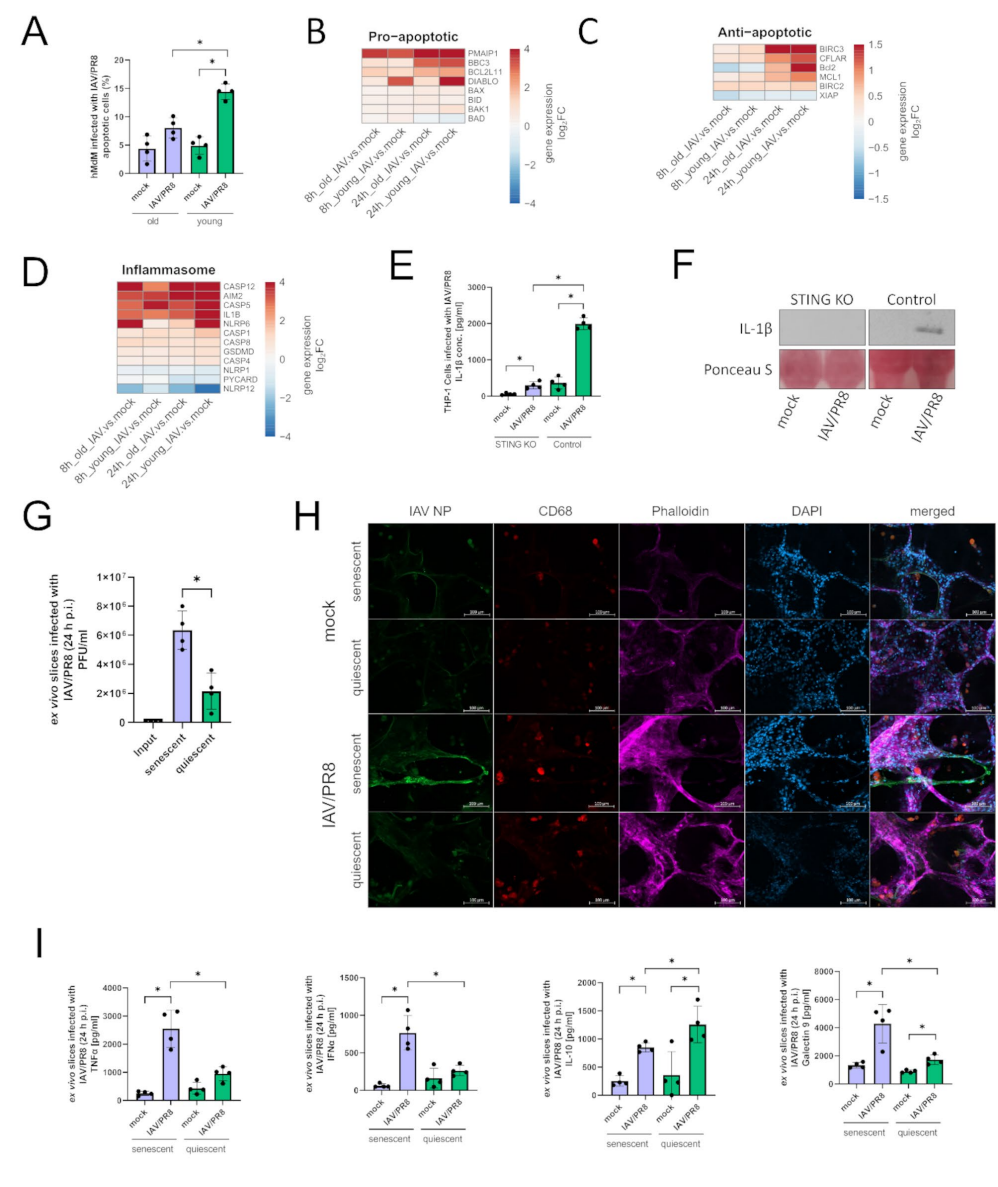
Apoptosis and inflammasome signaling pathways are altered in old macrophages. **(A)** Apoptotic cells were quantified via flow-cytometry. The results of four biological replicates in each group are shown. Significance was calculated with the Mann-Whitney U test (* *p* ≤ 0.05). **(B)** Heatmaps showing proapoptotic, **(C)** antiapoptotic genes **(D)** and genes of inflammasome signaling. Gene expression is presented over log2 fold change. At least one DEG per gene per condition had a significant log2 fold change. **(E)** Concentration of IL-1β was measured in the supernatants of infected THP-1 Dual and THP-1 Dual STING KO cells 24 h p.i. Results were confirmed by western blotting using the indicated antibodies. Significance was calculated with the Mann-Whitney U test (* *p* ≤ 0.05). **(F)** Supernatants of THP-1 cells were analyzed by western blot using the indicated antibody and staining. The experiment was repeated 4 times and blot shown as representative. **(G)** Viral titer of ex vivo lung slices was determined by standard plaque assay. Significance was calculated with the Mann-Whitney U test (* *p* ≤ 0.05). **(H)** Immunofluorescence staining of infected ex vivo lung slices stained with antibodies against IAV NP, CD68, phalloidin and DAPI. **(I)** Cytokines in the supernatants of senescent and quiescent ex vivo lung slices were measured. Significance was calculated with the Mann-Whitney U test (* *p* ≤ 0.05)

Distressed mitochondria and the consequent release of mtDNA in the cytoplasm are potential triggers of intrinsic apoptosis. Therefore, we measured the percentage of apoptotic vs. non-apoptotic hMdM after infection with IAV/PR8. Overall, aged hMdM displayed a lower number of apoptotic cells (Fig. 5A). Proapoptotic genes were upregulated as early as 8 h p.i., most strongly *PMAIP1*, which encodes the protein Noxa (Fig. 5B). Of interest was the upregulation of *DIABLO*, that occurred only in young hMdM. Anti-apoptotic genes were upregulated 24 h p.i. with a trend to a stronger upregulation also in young hMdM (Fig. 5C).

Several genes of inflammasome signaling pathways were upregulated in both age groups after infection (Fig. 5D). Of interest is the upregulation of *CASP5*, which so far has only been reported in LPS-stimulated immune cells [19]. Additionally, IL-1β was more strongly upregulated in young hMdM 24 h p.i. (Fig. 5D). We therefore also measured IL-1β in the supernatants of infected THP-1 cells. The THP-1 STING KO cells displayed a significant reduced production of IL-1β (Fig. 5E), which we also confirmed by western blot, indicating a role of STING in triggering the inflammasome signaling (Fig. 5F).

To further investigate age-dependent differences in the immune response to IAV, we induced premature senescence in human ex vivo lung slices by treating them with doxorubicin and subsequently infected them with IAV/PR8 [20]. The senescent ex vivo lung slices showed a higher viral load 24 h p.i. that could be verified with an increased intensity of the virus signal in the senescent slices by using immunofluorescence staining (Fig. 5G-H). The concentrations of TNFα, IFNα and the immunomodulatory lectin Galectin-9 were significantly higher, while the anti-inflammatory cytokine IL-10 was reduced in the senescent lungs (Fig. 5I).

In conclusion, our data indicates a decreased inflammasome activation due to low levels of STING resulting in downregulation of IL-1β in aged macrophages.

## Discussion

Acute pneumonia caused by IAV is linked to high morbidity and mortality rates worldwide. Especially old patients are at a higher risk for a severe course of disease [1–3]. In our study we investigated age-dependent modulations in the immune response of hMdM to infection with a IAV/PR8 and contemporary H1N1 IAV strain. Only the IAV/PR8 strain was able to infect and replicate successfully in hMdM (Fig. 1, Supplemental Fig. 3A). The ability of IAV to replicate in human macrophages is dependent on both the macrophage subset and IAV strain and Marvin et al. showed that the successful replication of IAV reduced the phagocytic capacities of macrophages in both murine and human cells [8, 9, 21]. However, the connection between viral replication and cytokine response has not been fully answered yet and needs further investigation [8, 22–24]. Since an immune response of hMdM was elicited only by a replicating IAV strain in our experimental setup, all further experiments were performed only with the IAV/PR8 strain.

In hMdM from aged donors we identified a reduced gene expression and production of interferons 24 h after infection, resulting in a higher viral titer (Fig. 1, Supplemental Fig. 3B). A similar deficiency of IFN production in response to infection with an H1N1 IAV strain was reported in aged human peripheral blood monocytes with otherwise intact cytokine response [25–27].

In order to identify underlying mechanisms of the impaired interferon production in the aged hMdM, mRNA sequencing was performed. Here we observed an upregulation of several genes contributing to DNA-sensing receptors in infected hMdM – including cGAS (Fig. 2). The activation of the cGAS-STING pathway by the IAV-induced release of mtDNA into the cytosol has been reported in several studies [17, 18]. Additionally, there is evidence of crosstalk between RNA-sensing and DNA-sensing pathways. This crosstalk is facilitated by the formation of complexes between STING and RIG-I/MAVS, which ultimately leads to the production of interferon [28]. Also, Holm et al. discovered a cGAS-independent activation of STING by enveloped RNA viruses such as IAV [29]. Strikingly, our data indicates that STING, encoded by *TMEM173*, was downregulated after virus infection and aged hMdM showed an overall reduced expression of STING (Fig. 2). To our best knowledge, we are the first to discover an age-dependent difference in gene expression of STING in hMdM, possibly leading to the diminished interferon production.

To further investigate the mechanism of STING-dependent IFN production, we deployed a THP-1 STING KO model. After infection, the THP-1 STING KO cells showed a delayed upregulation of the IFN activation pathway (Fig. 3). It is already known that a delayed type-I interferon production is associated with prolonged illness and a more severe course of disease in respiratory infections in mice [30–33].

By using a more complex human ex vivo lung model we could further show that the inhibition of STING by H151 decreases cytotoxicity and secretion of IFNα, TNFα and MCP-1 24 h after infection with IAV/PR8 (Fig. 3). Lv et al. reported an abrogation of the excessive immune response in a mouse infection model after applying H151, as well as a reduced activation of the NLRP3 inflammasome-associated production of IL-1β in aged human peripheral blood mononuclear cells (PBMCs), thus improving the course of disease and the outcome [17]. Likewise, Domizio et al. showed a protective effect of STING inhibition in mice infected with SARS-CoV-2 with reduced immunopathology in the lung [34]. Thus, STING is an important driver of inflammation during infection with respiratory RNA viruses; however, the regulation is dependent on cell type and experimental setup.

Few studies used human primary immune cells to investigate age-related changes and the immune response, instead relying on in vivo mice models and clinical data, which makes it difficult to distinguish between specific cell types. When studying macrophages in vitro, the heterogeneity of macrophage subpopulations should be taken into consideration. It is generally acknowledged that the immune response differs widely between macrophage subtypes [8, 9, 35]. While both AM and hMdM play an important role in the defense against IAV infection, their immune response is different [7, 35]. By using primary hMdM, we were able to systematically investigate age-related differences in a very specific macrophage subtype. The human ex vivo lung model then provided a complex infection model with numerous cell interactions under standardized in vitro conditions, thereby bridging the gap between mono-cell culture and in vivo studies.

Since we asked whether the transcriptional difference in STING genes was already caused by events further upstream, we wanted to assess mitochondrial functionality in hMdM during infection with IAV/PR8. In recent years the central role of mitochondria in viral infections has become increasingly apparent [36–38]. Important effector molecules produced by mitochondria are mtROS. Our data suggests oxidative stress happening in the mitochondria and an overall disruption of the mitochondrial network (Fig. 4). In the literature, there is evidence for both, a beneficial and detrimental role of mtROS during respiratory infections, also the activation of the NLRP3 inflammasome can be mediated by mtROS [39, 40]. Tal et al. reported increased RLR-signaling following the accumulation of ROS [41]. However, an excessive production of mtROS is associated with increased lung inflammation and mortality in respiratory infections [42, 43].

IAV is well known to induces lytic and non-lytic cell death in immune cells [44–46]. However, age-dependent differences in cell death induction are not well understood. When measuring apoptotic cells 24 h after infection with IAV/PR8, aged hMdM showed a lower number of apoptotic cells (Fig. 5). Additionally, the proapoptotic gene *DIABLO* was more strongly upregulated in young hMdM.

Finally, we induced senescence in human ex vivo lung slices using doxorubicin. This method is well-established as a model of cellular senescence [20, 47]. The senescent slices displayed a higher viral load and exaggerated proinflammatory immune response. An exacerbated inflammation is often observed in aged patients during respiratory infections, and it is generally accompanied by an increased mortality rate [48, 49].

Of interest are the increased levels of Galectin-9 in infected senescent lung slices. When applied exogenously, Galectin-9 induces apoptosis in mature T-cells [50]. In macrophages, Galectin-9 induces a proinflammatory phenotype [51]. During IAV infection, Galectin-9 directly binds to the surface of the virus, thus hindering its attachment [52]. There are, to the best of our knowledge, no studies yet that describe age-dependent differences in Galectin-9 expression. In the context of infectious research, Galectin-9 could be a promising molecule for further investigations.

In summary our study provides a systematic comparison of the immune response to IAV infection in aged and young hMdM. We identified an impaired interferon production in aged hMdM leading to a higher susceptibility to infection. An age-dependent decrease of STING expression provides a possible explanation for the diminished immune response. In conclusion, our data adds valuable input to our understanding of age-related changes in macrophage function during Influenza Virus infections.

## Materials and methods

### Virus strains

IAV strains were propagated in Madin-Darby canine kidney (MDCK) cells cultivated in minimum essential medium (MEM, Thermo Fisher Scientific, Waltham, USA) with 10% fetal bovine serum (FBS, PAN Biotech, Aidenbach, Germany) and 1% penicillin/streptomycin (P/S, Lonza, Basel, Schweiz). Supernatant was collected when cytopathic effect was apparent, centrifuged at 4 °C and aliquots were stored at -80 °C. Virus titer was determined by standard plaque assay [53].

### Monocyte isolation

PBMCs were isolated from commercially obtained buffy coats using Histopaque-1077 (Sigma Aldrich, Taufkirchen, Germany) and density centrifugation. Cells from donors ≥ 65 years were considered as aged and ≤ 25 years as young. After washing cells were plated in Roswell Park Memorial Institute (RPMI, Thermo Fisher Scientific) medium and cultured at 37 °C, 5% CO_2_ for 3 h. After attachment of monocytes, cells were washed and cultivated in RPMI, 10% human serum albumin (HSA, PAN Biotech), 1% P/S and 0.01% granulocyte-macrophage colony-stimulating factor (GM-CSF, Thermo Fisher Scientific) for 7 days to stimulate differentiation to hMdM [20]. Medium was changed every 2–3 days.

### THP-1 cell culture and assays

THP-1 cells were cultured in RPMI with 10% FBS, 25 mM HEPES (Thermo Fisher Scientific) and 1% P/S. For differentiation cells were incubated with 50 ng/ml Phorbol 12-myristate 13-acetate (PMA, Sigma-Aldrich) for 3 h. After attachment, cells were washed with Dulbecco´s Phosphate Buffered Saline (DPBS, Thermo Fisher Scientific) and cultured in complete media for 3 days.

For detection of pathway induction in THP-1 Dual and THP-1 STING KO Dual cells (Thermo Fisher Scientific) corresponding assays were performed according to manufacturer´s instructions (Supplemental Fig. 2A).

For redox assays, the fluorescence of THP-1 cells expressing *ro-GFP2*, *roGFP2-Su9* and *roGFP2-OTC* was measured at *λ*_ex_ of 405 nm and 488 nm and calculated as relative fluorescence intensity (RFI).

### Human ex vivo precision-cut lung slices

Human lung tissues were provided by the Department of Cardiothoracic Surgery, Jena University Hospital – Friedrich Schiller University of Jena. The preparation of ex vivo lung slices was done as previously described [54].

Briefly, the human lung lobe was filled with a mixture of 4% top vision low-melting point agarose (Thermo Fisher Scientific) and Dulbecco´s Modified Eagle´s Medium (DMEM)/F12 w/o. phenol-red (Thermo Fisher Scientific) at a 1:1 ratio and instilled into the tissue. After solidification of the agarose, the tissue was sliced into cubes and cut into 300 μm slices by a Vibratome (Leica – VT1200S, Leica Biosystems, Germany). Slices were transferred to 12 well plates with 1 ml DMEM/F12 and 1% P/S and stored at 37 °C with 5% CO_2_ (Supplemental Fig. 2B).

After 24 h of adjustment in the well plate, slices were infected with a concentration of 5 × 10^5^ PFU/ml of IAV/PR8 diluted in Infection-DPBS for 2 h at 37 °C, 5% CO_2_. After washing, fresh medium was added, and slices were monitored for 24 h.

### In vitro infections

The in vitro infections were performed with the IAV strains influenza A virus/IAV/H1N1/Puerto Rico/8/1934 (IAV/PR8) and influenza A virus/IAV/H1N1/Jena/84/2016 (IAV/J84).

The virus solution was diluted in RPMI with 10% HSA at a multiplicity of infection (MOI) 1. Medium was removed, cells were washed once with DPBS and incubated in pre-warmed infection medium for 24 h. Supernatants were collected, and the viral load was determined by standard plaque assay.

### Induction of senescence

Human ex vivo lung slices were treated with 400 nM doxorubicin (TOCRIS, Bristol, UK) for 24 h. The slices were then incubated for 5 d to let the senescence-associated phenotype develop.

### Stimulation with STING inhibitor

Before the infection, ex vivo slices were treated with 5 µM of the specific STING inhibitor H151 (Invivogen, San Diego, USA) diluted in the culture medium for 24 h. The inhibitor (5 µM) was also added to the medium during the infection.

### Immunofluorescence staining

Cells were cultured on coverslips, fixed with 4% paraformaldehyde (PFA, Sigma Aldrich) for 15 min at 37 °C and stored at 4 °C in DPBS until staining. Ex vivo slices were fixed for 1 h at 37 °C. Samples were permeabilized with 0.1% Triton-X (Roth, Kralsruhe, Germany) and blocked with 3% bovine serum albumin (BSA, Roth) in DPBS. The samples were incubated with IAV NP antibody (abcam, Cambridge, UK) and CD68 (Thermo Fisher Scientific) diluted 1:200 in blocking buffer overnight at 4 °C. Staining of mitochondria was performed before fixation by incubating samples with 100 nM MitoTracker Red and MitoTracker Green respectively (Thermo Fisher Scientific) for 30 min.

The following day, samples were incubated with anti-rabbit and anti-mouse secondary antibodies (Jackson Immuno Research, West Grove, Pennsylvania, USA) and Alexa Fluor Phallodin stainings (Thermo Fisher Scientific) diluted 1:500 and 1:400 in blocking buffer respectively for 1 h at RT. Cells were mounted with Fluoromount-G (Southern Biotech, Birmingham, USA) and microscopy was performed at an AxioObserver Z.1 + Apotome 2 (Zeiss, Jena, Germany). The images were analyzed using the Zen software (Zen Pro v3.3).

### Flow cytometry analysis

Detachment of macrophages was done by incubating 30 min with Accutase (Sigma Aldrich) and gentle scraping. For macrophages characterization, the cells were incubated with Fixable Viability Staining 780 (BD Biosciences, Franklin Lakes, New Jersey, US) for 15 min at RT. After washing, cells were fixed and permeabilized with BD Fixation/Permeabilization solution (BD Biosciences) at 4 °C for 20 min. The samples were then stained with an antibody against IAV NP (abcam) at a dilution of 1:100 at 4 °C for 30 min.

For quantification of mtROS cells were incubated with 3 µM MitoSOX (Thermo Fisher Scientific) dye at 37 °C for 20 min. ATP measurement and Apoptosis quantification were performed with the BioTracker ATP-Red Live Cell Dye (Sigma Aldrich) and BD Pharmingen FITC Annexin V Apoptosis Detection Kit I (BD Biosciences) according to the manufacturer’s instructions. To discriminate live cells, samples were incubated with 7-AAD (BD Biosciences) for 10 min.

Samples were analyzed using a FACS Symphony A1 (BD Biosciences) and the software FlowJo v10.8.1.

### Cytokine and LDH quantification

Cytokine quantification of supernatants was performed with the LEGENDplex Human Anti-Virus and LEGENDplex Human Inflammation Panel 1 Kits (BioLegend, San Diego, USA) according to the manufacturer’s instructions. Samples were analyzed using a FACS Symphony A1 (BD Biosciences) and the Qognit software v2023-02-15 (BD Biosciences).

LDH quantification was performed with the CyQUANT LDH Cytotoxicity Assay according to the manufacturer´s instructions (Thermo Fisher Scientific).

### Protein extraction and ELISA

Cells were lysed in cell extraction buffer (Cell Signaling, Danvers, Massachusetts, US). Protein concentrations of samples were determined using the Micro BCA Protein Assay Kit (Thermo Fisher Scientific). Quantification of cGAS and cGAMP (Thermo Fisher Scientific) was performed according to the manufacturer’s instructions. Samples were analyzed using a Tecan microplate reader “Infinite 200 Pro” (Tecan Life Sciences, Zürich, Switzerland).

### Subcellular fractioning

For total protein, extracted cells were lysed in SDS lysis buffer containing 20 mM Tris and 1% SDS (v/v) and boiled at 95 °C for 15 min.

To seperate cytosolic fraction, cells were incubated with digitonin lysis buffer containing 50 mM HEPES, 150 mM NaCl, 30 µg/ml digitonin for 10 min at 4 °C. For separation of the mitochondrial fraction, cells were subsequently lysed by NP-40 lysis buffer containing 50 mM Tris, 150 mM NaCl, 1 mM EDTA, % NP-40 (v/v) and 1% glycerol (v/v) and incubated for 10 min at 4 °C. To obtain the nuclear fraction, cells were lysed in SDS lysis buffer at 95 °C for 15 min.

### Western blot

Proteins were run on SDS-PAGE and blotted onto polyvinylidene fluoride (PVDC, Thermo Fisher Scientific) membranes. Supernatants from THP-1 and THP-1 STING KO were boiled for 10 min with Laemmli Protein Sample Buffer (BioRad, CA, USA). Proteins were transferred to nitrocellulose membranes (Cytivia, Amersham, UK) and were stained with Ponceau S (Sigma Aldrich) as a loading control. Membranes were blocked with 5% milk (v/v) in TBS-T for 1 h and incubated with primary antibodies against GAPDH (Proteintech, San Diego, CA, USA), Cleaved-IL-1β (Cell Signaling, MA, USA) and TFAM (Proteintech) diluted 1:1000 in blocking buffer at 4 °C overnight. On the next day, membranes were incubated with horseradish peroxidase (HRP)-conjugated anti-rabbit and anti-mouse IgG secondary antibody (Bio-rad, Hercules, USA) diluted 1:1000 in blocking buffer and with IL-1β peroxidase-labeled anti-rabbit IgG (H + L, 1:10000, Vector Laboratories). Immobilon Western chemiluminescent HRP substrate (Merck Millipore, Darmstadt, Germany) was used for visualization and pictures were taken on a FusionFX imaging device (Vilber, Collégien, France) and UVITEC Mini HD9 (Uvitec, Cambridge, UK).

### DNA isolation and qPCR

DNA was isolated using the DNeasy Blood & Tissue Kit (QIAGEN, Hilden, Germany) according to the manufacturer´s protocol. DNA concentration was determined using a NanoDrop spectrophotometer ND-1000 (Peqlab/VWR, Radnor, USA). For qPCR assays Maxima SYBR Green qPCR Master Mix (Thermo Fisher Scientific, Germany) and Rotor-Gene Q (QIAGEN) were used. Primers were acquired from metabion (metabion, Planegg, Germany) (KCNJ10 forward GCGCAAAAGCCTCCTCATT, reverse CCTTCCTTGGTTTGGTGGG; MT-D-Loop forward CATAAAGCCTAAATAGCCCACACG, reverse CCGTGAGTGGTTAATAGGGTGATA).

### RNA isolation and mRNA sequencing

Cells were lysed in RLT buffer and total RNA was isolated using the RNeasy Mini Kit (QIAGEN). RNA concentration was determined by a NanoDrop spectrophotometer ND-1000 (Peqlab, VWR, Germany). Transcriptome sequencing and preparation of the RNA library was carried out by Novogene Co., LTD (Beijing, China), using the Illumina platform Novaseq 6000 S4 flowcell V1.0. The technique is based on the mechanism of sequencing by synthesis (SBS) and the PE150 strategy (NEB Next Ultra RNA Library Prep Kit). Heatmaps were generated using R (v3.6.3) (R Foundation for Statistical Computing, https://www.R-project.org/).

### Transmission electron microscopy

For fixation the lung tissue slices were incubated in freshly prepared modified Karnovsky fixative (4% w/v paraformaldehyde, 2.5% v/v glutaraldehyde in 0.1 M sodium cacodylate buffer pH 7.4) for 24 h at RT. The samples were washed 3 times with 0.1 M sodium cacodylate buffer (pH 7.4) and post-fixed with 2% w/v osmiumtetroxide for 1 h at RT. During the following dehydration in ascending ethanol series post-staining with 1% w/v uranylacetate was performed. Afterwards embedding the samples in epoxy resin (Araldite) they were cut in ultrathin sections (thickness 70 nm) using a Leica Ultracut S (Leica, Wetzlar, Germany). Mounting was performed on filmed Cu grids, and samples stained with lead citrate. The sections were analyzed in a transmission electron microscope (EM 900, Zeiss, Oberkochen, Germany) at 80 kV and magnifications of 3000x to 85,000x and pictures taken with a 2 K slow scan CCD camera (TRS, Moorenweis, Germany).

### Statistical analysis and illustrations

Statistical analysis was done with GraphPad Prism 9 using Mann-Whitney U test, Kruskal-Wallis test, Student´s *t* test and One-way Anova. Non-parametric tests were chosen when comparing < 6 biological replicates per group and parametric tests when ≥ 6 biological replicated per group were compared. The figures were created with BioRender.com.

## Electronic supplementary material

Below is the link to the electronic supplementary material.

**Supplementary material 1**: **Supplemental Fig. 1**. (A) Viral replication in hMdM was determined by standard plaque assay 24 h p.i. The experiment was performed with four young and old donors each. Significance was calculated with Mann-Whitney U test. (B) Venn diagram of DEG in infected hMdM at timepoints 8 h and 24 h p.i. (C, D) Volcano plot displaying up and downregulated gene when comparing infected old to infected young hMdM (8 h and 24 h p.i.). (E) KEGG Enrichment analysis of hMdM (IAV vs. mock) at timepoints 8 h and 24 h p.i. **Supplemental Fig. 2**. (A) Schematic representation of reporter genes for IFN and NFκB pathway activation in THP-1 Dual Cells. (B) Schematic representation of the preparation of human ex vivo slices. (C) Immunofluorescence staining of ex vivo slices infected with IAV/PR8 (24 h p.i.) stained with antibodies against IAV NP, CD68, phalloidin and DAPI. **Supplemental Fig. 3**. (A) Graphical abstract of the study. (B) Overview of all methods used in this study.

Supplementary Material 2

## Data Availability

No datasets were generated or analysed during the current study.
