# Supplementary material for: Age-related STING suppression in macrophages contributes to increased viral load during influenza a virus infection
Source: Immun Ageing. 2024 Nov 14;21:80. doi: 10.1186/s12979-024-00482-9 (PMC11562583; doi:10.1186/s12979-024-00482-9)
Supplement: Supplementary file 1 — Supplementary material 1: Supplemental Fig. 1. (A) Viral replication in hMdM was determined by standard plaque assay 24 h p.i. The experiment was performed with four young and old donors each. Significance was calculated with Mann-Whitney U test. (B) Venn diagram of DEG in infected hMdM at timepoints 8 h and 24 h p.i. (C, D) Volcano plot displaying up and downregulated gene when comparing infected old to infected young hMdM (8 h and 24 h p.i.). (E) KEGG Enrichment analysis of hMdM (IAV vs. mock) at timepoints 8 h and 24 h p.i. Supplemental Fig. 2. (A) Schematic representation of reporter genes for IFN and NFκB pathway activation in THP-1 Dual Cells. (B) Schematic representation of the preparation of human ex vivo slices. (C) Immunofluorescence staining of ex vivo slices infected with IAV/PR8 (24 h p.i.) stained with antibodies against IAV NP, CD68, phalloidin and DAPI. Supplemental Fig. 3. (A) Graphical abstract of the study. (B) Overview of all methods used in this study. [file 12979_2024_482_MOESM1_ESM.docx]

**Supplemental Figure 1. (A)** Viral replication in hMdM was determined by standard plaque assay 24 h p.i.. The experiment was performed with four young and old donors each. Significance was calculated with Mann-Whitney U test. **(B)** Venn diagram of DEG in infected hMdM at timepoints 8 h and 24 h p.i.. **(C, D)** Volcano plot displaying up and downregulated gene when comparing infected old to infected young hMdM (8 h and 24 h p.i.). **(E)** KEGG Enrichment analysis of hMdM (IAV vs mock) at timepoints 8 h and 24 h p.i.

**Supplemenl Figure 2. (A)** Schematic representation of reporter genes for IFN and NFκB pathway activation in THP-1 Dual Cells. **(B)** Schematic representation of the preparation of human *ex vivo* slices. **(C)** Immunofluorescence staining of *ex vivo* slices infected with IAV/PR8 (24 h p.i.) stained with antibodies against IAV NP, CD68, phalloidin and DAPI.

**Supplemental Figure 3. (A)** Graphical abstract of the study. **(B)** Overview of all methods used in this study.
