# Supplementary material for: Age-related STING suppression in macrophages contributes to increased viral load during influenza a virus infection
Source: Immun Ageing. 2024 Nov 14;21:80. doi: 10.1186/s12979-024-00482-9 (PMC11562583; doi:10.1186/s12979-024-00482-9)

Supplemental Figure 1.

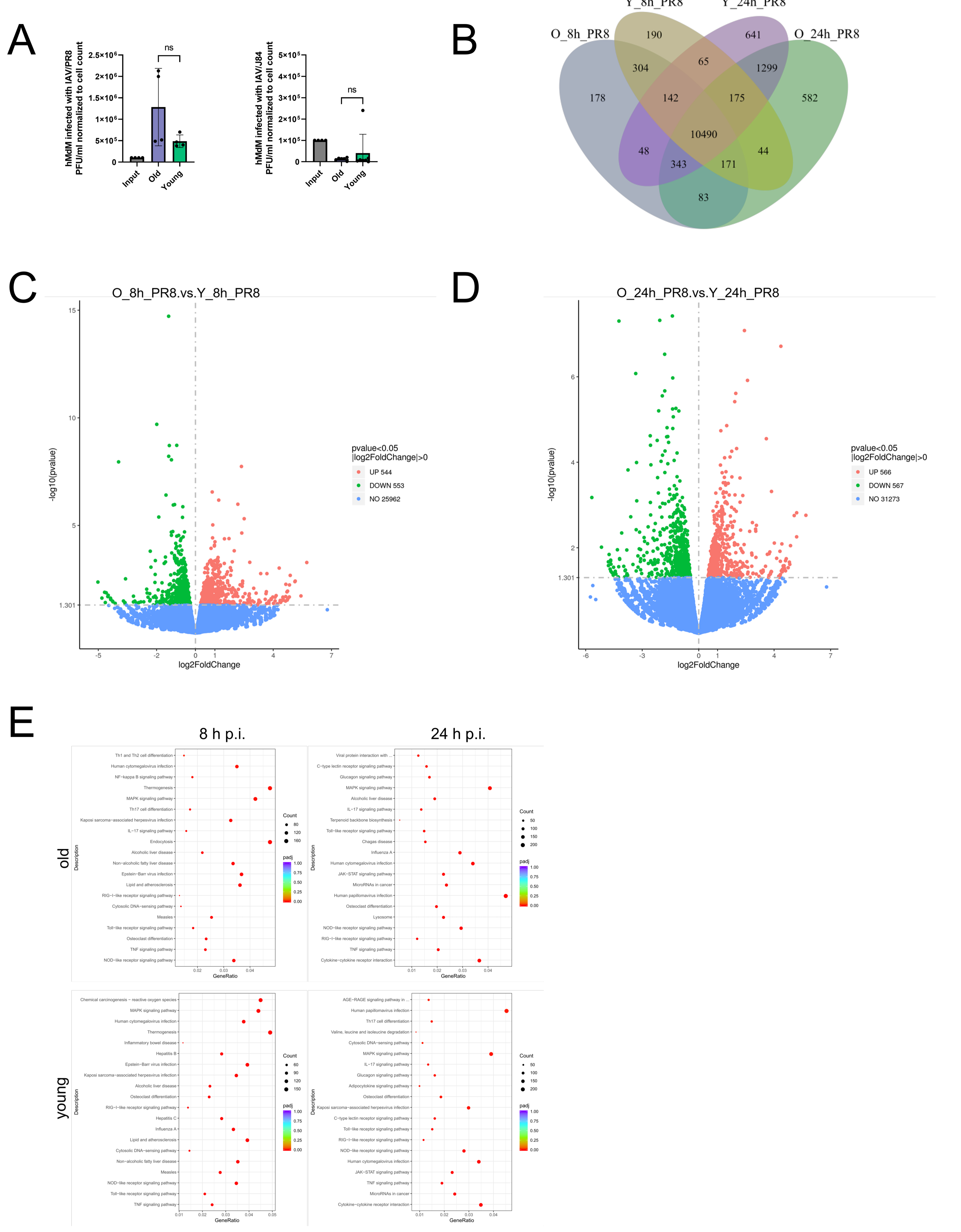

Supplemental Figure 2.

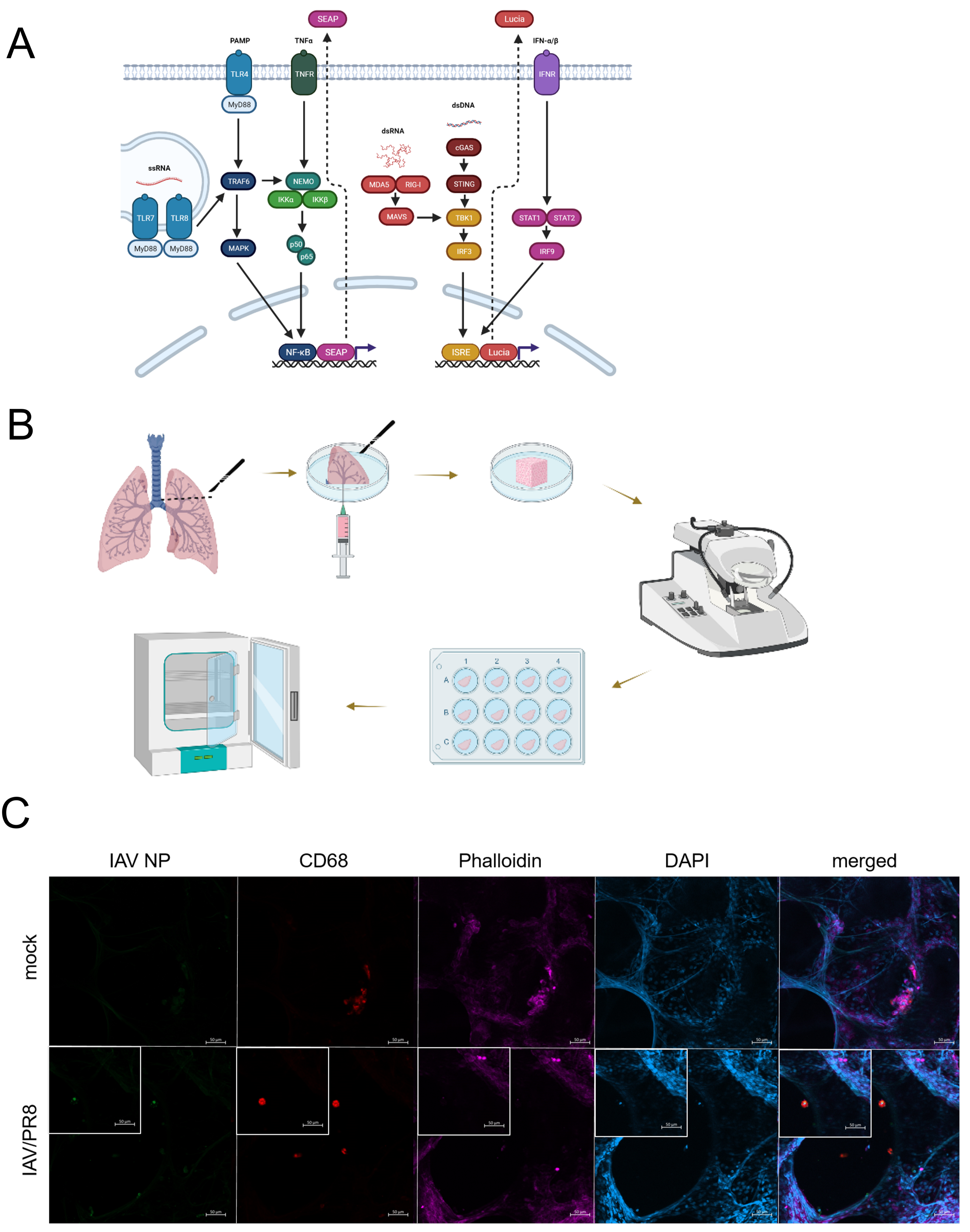

Supplemental Figure 3.

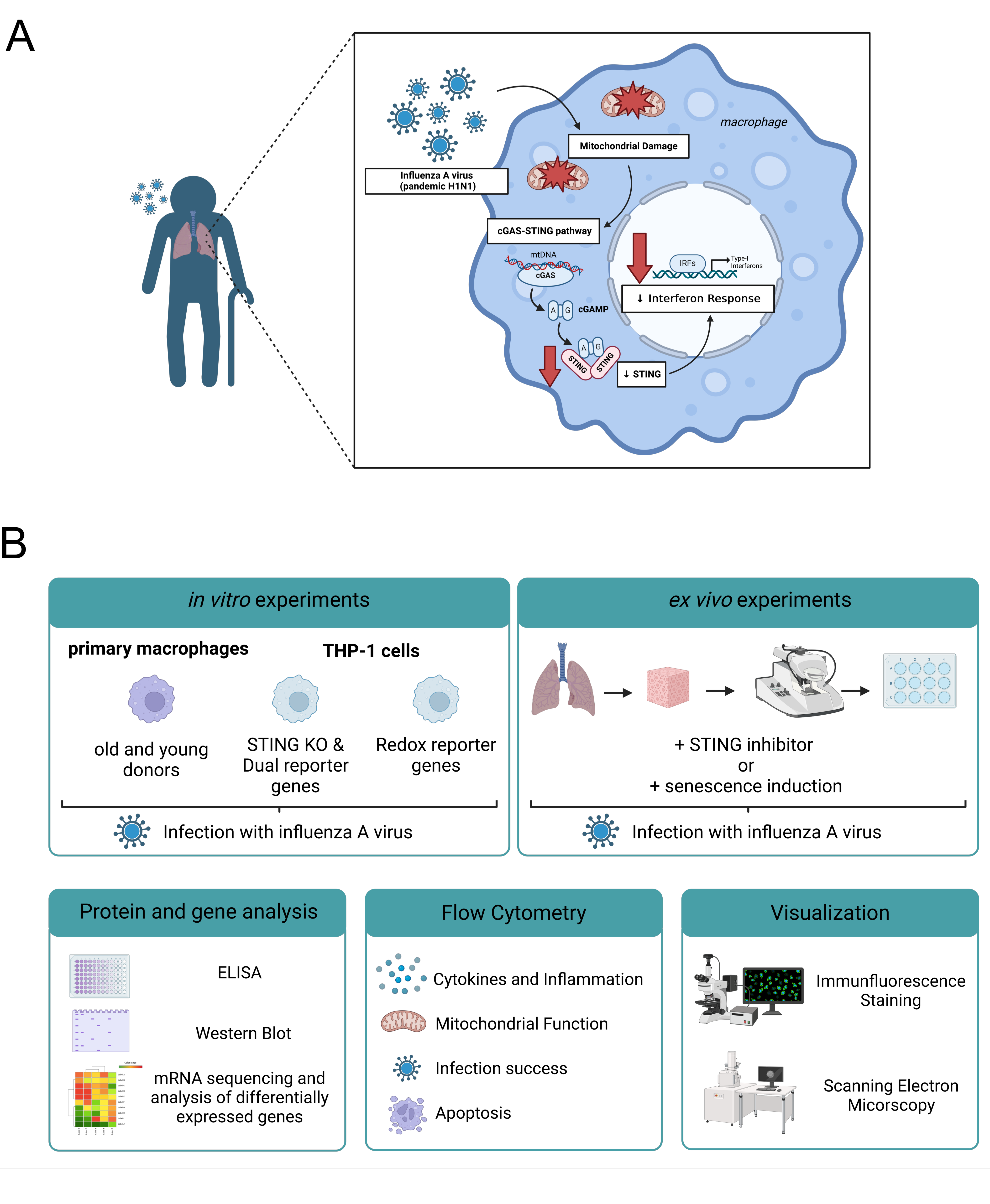

Supplement: Supplementary file 2 — Supplementary Material 2 [file 12979_2024_482_MOESM2_ESM.pdf]
